# Nervous Diseases, Arising from Liver and Stomach Complaints, Low Spirits, Indigestion, Gout, and Disorders Produced by Tropical Climates; with Cases

**Published:** 1844-07

**Authors:** 


### Art. XXIII.

1. *Nervous Diseases, arising from Liver and Stomach Complaints, Low Spirits, Indigestion, Gout, and Disorders produced by Tropical Climates; with Cases.* By G. R. Rowe, m.d. f.s.a. *Sixth Edition.—London*, 1843. 8vo, pp. 184.
2. *On some of the most Important Disorders of Women.* By G. R. Rowe, m.d. f.s.a.—*London*, 1844. 8vo, pp. 120.

The title and recent publication of the second of these books naturally led us, in the exercise of our critical duties, to procure and read it; and its examination led to the perusal of the first work, as we found it stated in the new publication, that the doctrines inculcated in it would be found more fully exposed in the old. We were, moreover, curious to see the qualities of a medical book which, in these times, could command a sale of six editions. The result of our examination of both works is a judgment so unfavorable, that we should much regret the valuable time lost in the task, had we not been thereby enabled to warn our readers against the danger of committing the same mistake which we have done. In truth, the volumes, although bearing the semblance of professional treatises, are really written for the public; and they can only be regarded, by the impartial critic, as identical in their character and object, with the literary productions of those eminent scientific men whose benevolent purposes in regard to suffering humanity are set forth in the advertising columns of all our morning and Sunday papers. Dr. Rowe's books are, obviously, advertisements calculated and destined to inform all and sundry whom it may concern, that at "Chigwell, Essex, and Golden Square, London," (the place of date of the preface and dedication,) they will be most effectually cured of all their ails. The great body of both volumes consists of cases of the most popular diseases, written in a form intelligible to all, and portraying in glaring colours the horrors and dangers of sickness, and the potency of the therapeutics employed by the author to restore the all-prized blessings of health. In both books, out of the whole forty-nine or fifty cases, we think there is no one in which the patient is left uncured or not marvellously benefited.

We are the more decided in our opinion of the object of the author of these works, as we cannot believe that Dr. Rowe can be so ignorant or simple as not to know that they convey not one particle of information not already possessed by every medical tyro; and because he does not profess to teach the lay readers to cure themselves. Far from it: no opportunity is lost for pointing out the vast importance of professional discrimination and skill in the management of the diseases; and the invariably happy event of the author's own treatment tells silently, but with irresistible effect, that, although there may possibly be pools of healing elsewhere, the veritable Bethesda may with confidence be sought within the classical parallelogram of the Square of Gold, or amid the happy shades of Chigwell.

And yet, after all, Dr. Rowe's books are not positively bad, but only utterly useless. His practice is even, on the whole, good; and many of his doctrines are sound enough. He is, however, much too exclusive. The source of all his maladies lies in what he invariably calls the *prima via*, (on the classical principle, doubtless, of the alderman's daughter, who grumbled at the too numerous *omnibi* that passed her window;) and he strongly inculcates temperance, exercise, and the use of purgatives. But who knows not the value of these measures, and who does not prescribe them?

We said, not long since, in this Journal, that it was a comfort to well-educated and high-minded physicians to find that these popular charmers of the public almost invariably demonstrated, with their own hands, their unfitness for the ranks they had abandoned. The works before us form no exception to this general rule: they prove the total incapacity of the author to write his mother tongue; and justify the inference, that his scientific knowledge bears some proportion to his literary proficiency. Authors of this class seem to have an instinctive feeling of the value of their literary efforts, by the kind of critics they choose to be judged by. Accordingly, we find prefixed to one of the works before us no less than twelve huge pages of small type, blazoning the "*Opinions of the Public Press*," in a series of extracts of a commendatory nature, from the following high authorities: the Times, Morning Advertiser, Argus, Æra, Naval and Military Gazette, Essex Herald, Chelmsford Chronicle, Bucks Herald, Court Journal, Sunday Times, City Chronicle, Weekly Messenger, Indian Review, Cheltenham Chronicle, Essex Standard, Western Watchman, and London News!!! The only *medical* critics immortalized in this list are the Lancet and Medical Times. It is hardly necessary to mention to our readers the *means* whereby such a galaxy of popular glory can alone be created in these degenerate utilitarian days.

In justification of our assertion, that Dr. Rowe cannot write English, we give a few specimens, taken at random from his books: every page might supply similar evidence.

"........Temperate modes of life, and which must be regarded as the grand *coadjutors to the enjoyment of health, as well as to the annihilation of diseases.*" (Nervous Diseases, p. 12.) "The contractile state of the pupil of the eye......; the increased secretion from the lacrymal glands, when any irritating substance is applied to the eyes, *are decided and merciful convictions* of that principle of self-preservation." (Ib. p. 19.) "Primitive and most *potent symptoms.*" (Ib. p. 27.) "The mind is *attended with great despondency.*" (Ib. p. 95.) "To assist the removal of *costiveness,* patients should be induced to visit a place *appropriated for that purpose* daily," &c. (Ib. p. 103.) "The foundation of his disease was laid by *an innate desire for spirits.*" (Ib. p. 113.) "During the nights and in the mornings she talked incoherently, but throughout the day she had intervals of *sensibility.*" (Ib. 122.) "The *liver* either becoming *hepatized* or obstructed," &c. (Ib. p. 153.)

"From her first merging into womanhood, the pains and penalties inflicted upon her [woman] *the danger and modest solicitude peculiar to her sex alone.*" (Disorders of Women, p. 5.) "Yet how wantonly is it [health] sported with, *tampered,* and abused." (Ib. p. 6.) "....the increasing *emotions of the nervous system.*" (Ib. p. 11.) "....for all physiologists concur in the powerful dominion of that viscus [the uterus] over the *sympathies and various classes of animal life.*" (Ib. p. 23.) "....it is evident that *in proportion to its quantity* would the size of the fœtus *depend.*" (Ib. p. 27.) "....his must *prove as a powerful evidence.*" (Ib. p. 36.) "....the domestic habits of women tend much to produce inactivity and *generate muscular prostration.*" (Ib. p. 42.) "Can anything appear more *inconsistent than to witness* the well-protected feet of men," &c. (Ib., p. 52.) "Vicissitudes of climate and *transition of situation.*" (Ib. p. 55.) "But *every age has vied with each other.*" (Ib. p. 75.) "Enable that organ [the stomach] to *perform its natural powers.*" (Ib. p. 78.) &c. &c. &c. &c.

